# Genetic deletion of dectin-1 does not affect the course of murine experimental colitis

**DOI:** 10.1186/1471-230X-12-33

**Published:** 2012-04-16

**Authors:** Sigrid EM Heinsbroek, Anneke Oei, Joris JTH Roelofs, Shobhit Dhawan, Anje te Velde, Siamon Gordon, Wouter J de Jonge

**Affiliations:** 1 Tytgat Institute for Liver and Intestinal Research, Academic Medical Center, University of Amsterdam, AMC, Amsterdam, The Netherlands; 2 Department of Medical Microbiology, Academic Medical Center, University of Amsterdam, AMC, Amsterdam, The Netherlands; 3 Department of Pathology, Academic Medical Center, University of Amsterdam, AMC, Amsterdam, The Netherlands; 4 Sir William Dunn School of Pathology, University of Oxford, Oxford, UK; 5 Tytgat Institute for Liver and Intestinal Research, Academic Medical Center, Meibergdreef 69-71, 1105, BK Amsterdam, The Netherlands

**Keywords:** Dectin-1, Macrophages, Colitis, Innate immunity, Fungi, Intestine

## Abstract

**Background:**

It is believed that inflammatory bowel diseases (IBD) result from an imbalance in the intestinal immune response towards the luminal microbiome. Dectin-1 is a widely expressed pattern recognition receptor that recognizes fungi and upon recognition it mediates cytokine responses and skewing of the adaptive immune system. Hence, dectin-1 may be involved in the pathogenesis of IBD.

**Methods:**

We assessed the responses of dectin-1 deficient macrophages to the intestinal microbiota and determined the course of acute DSS and chronic *Helicobacter hepaticus *induced colitis in dectin-1 deficient mice.

**Results:**

We show that the mouse intestinal microbiota contains fungi and the cytokine responses towards this microbiota were significantly reduced in dectin-1 deficient macrophages. However, in two different colitis models no significant differences in the course of inflammation were found in dectin-1 deficient mice compared to wild type mice.

**Conclusions:**

Together our data suggest that, although at the immune cell level there is a difference in response towards the intestinal flora in dectin-1 deficient macrophages, during intestinal inflammation this response seems to be redundant since dectin-1 deficiency in mice does not affect intestinal inflammation in experimental colitis.

## Background

Pattern recognition receptors (PRR) are important for host recognition of microorganisms. Various groups of PRR are known which include the Toll-like receptors (TLR), NOD-like receptors and C-type lectin-like receptors (CLR). From many studies investigating TLR and NLR receptor function in the intestine it became clear that interaction between the intestinal microbiome and PRRs expressed in the intestine is important in modifying the intestinal immune system. TLRs and NLRs have been shown to be involved in regulating epithelial barrier function, secretion of antimicrobial peptides, IgA production and secretion into the intestinal lumen, lymphoid tissue development and T cell function [1,2]. Furthermore, Myd88, several TLRs, NOD2 and NLPR3 deficient mice have all been shown to be more susceptible to Dextran Sulphate Sodium (DSS) induced colitis [3-9]. From patient studies it is clear that various mutations in PRR are associated with IBD [10-13].

The current research on the host interactions with the microbiota mainly focuses on the bacterial component, however fungi are also present in the intestine [14], and interact with pattern recognition receptors, mainly CLRs like dectin-1, mannose receptor and DC-SIGN [15]. More than 55% of Crohn's Disease (CD) patients make antibodies against the mannan component of fungi compared to only 8% of healthy individuals and antibody titre is thought to be related to disease severity [16]. CD patients also make antibodies against other fungal components such as chitin and β-glucans [16,17]. Therefore fungi and the PRR recognising them may play a role in intestinal homeostasis.

CLRs are expressed in the intestine however, the interaction between CLRs and the intestinal microbiota is not well studied and in this paper we focus on dectin-1. Dectin-1 binds β-glucans found on fungi and upon recognition mediates phagocytosis and various cytokine responses including TNF-α and IL-10 production [18-21]. Co-stimulation of dectin-1 and TLR2-TLR6 has been shown to increase stimulation for TNFα, IL-12 and IL-2 production [19,20,22]. Dectin-1 has also been shown to induce dendritic cell maturation and direct T cell Th17 responses directly linking innate and adaptive immunity [23,24]. However, little is known about the role of dectin-1 in maintaining intestinal homeostasis. Dectin-1 is highly expressed in the intestine [25] and humans with CD have also been shown to have increased numbers of dectin-1 expressing inflammatory cells in the intestine compared with healthy individuals [26]. Together this implies that dectin-1 may play a role in intestinal immunity.

In order to establish the role of dectin-1 in intestinal immunity we determined dectin-1 expression in mouse colon and investigated responses towards the intestinal microbiota by macrophages deficient in dectin-1. Furthermore, we induced colitis in dectin-1 deficient mice using DSS colitis and a *Helicobacter hepaticus *induced colitis model to investigate the role of dectin-1 in intestinal inflammation.

## Methods

### Animals

Breeding colonies of C57BL/6 and C57BL/6 dectin-1^-/- ^(A kind gift from Gordon D. Brown) mice were housed and maintained under specific pathogen free conditions in our animal facility at the Academic Medical Center in Amsterdam. Animals were kept and handled in accordance with the guidelines of the Animal Research Ethics Committee of the University of Amsterdam.

### Determination of fungi

1 gram of mouse faeces was dissolved in PBS and plated on Sabouraud agar plates containing penicillin and gentamicin. The colonies were identified using an Auxacolor identification kit (Bio-rad).

### Colitis experiments

Animals were sex-matched and between 8 and 12 weeks of age at the time of study. For DSS colitis experiments 1,5% (w/v) DSS (TdB Consultancy, Uppsala, Sweden) was added to the drinking water for 7 days. Body weight was recorded daily, and weight loss on day 7 as compared with day 0 was calculated. On day 7 of DSS administration, animals were killed and organs collected. The wet weight of spleen and colon was recorded together with the total length of the colon. Colon weight per 5 cm was used as a disease parameter.

For *Helicobacter hepaticus *induced colitis *H. hepaticus *NCI-Frederick isolate 1A (strain 51449; American Type Culture Collection) was grown and maintained as previously described [27]. Mice were inoculated intragastrically on days 0, 2, and 4 with 5 × 10^7^-2 × 10^8 ^CFU *H. hepaticus*. Anti-IL-10R monoclonal antibody was produced and characterized as previously described [28]. Antibody treatment was commenced on the day of the first inoculation with *H. hepaticus*, and mice received weekly i.p. injections of 1 mg anti-IL-10R for the duration of the experiment. After 4 weeks animals were killed and blood, spleen, liver, cecum and colon collected.

### Histochemistry

The longitudinally divided colons were rolled, fixed in 4% formalin and embedded in paraffin for routine histology. An experienced pathologist evaluated formalin-fixed haematoxylin tissue sections microscopically, in a blinded fashion. Colons were evaluated, and graded from 0 to 4 as an indication of incidence and severity of inflammatory lesions based on the extent of the area involved, the number of follicle aggregates, oedema, fibrosis, hyperplasia, erosion/ulceration, crypt loss and infiltration of granulocytes and mononuclear cells as indicated in Table 1. The total inflammation score was calculated as the average score of the above.

**Table 1:** Colitis total inflammation score

score	0	1	2	3	4
**Area involved**	0%	1-10%	10-25%	25-50%	> 50%
**follicles**	Normal (0-1)	Minimal (2-3)	Mild (4-5)	Moderate (6-7)	Severe (> 7)
**edema**	Absent	Minimal	Mild	Moderate	Severe
**fibrosis**	Absent	Minimal	Mild	Moderate	Severe
**Erosion/ulceration**	0%	1-10%	10-25%	25-50%	> 50%
**Crypt loss**	0%	1-10%	10-25%	25-50%	> 50%
**granulocytes**	Normal	Minimal increase	Mild increase	Moderate increase	Severe increase
**Mononucl. cells**	Normal	Minimal increase	Mild increase	Moderate increase	Severe increase

The total inflammation score was determined by the average score of the following criteria: area involved, the number of follicle aggregates, oedema, fibrosis, hyperplasia, erosion/ulceration, crypt loss and infiltration of granulocytes and mononuclear cells.

### Immunohistochemistry

Frozen sections of mouse colon (10 μm) were processed for immunohistochemistry as previously described [25]. Briefly, slides were ethanol fixed and blocked in 10% normal rabbit serum for 10 min. The slides were drained and incubated with purified mAb 2A11 or the rat isotype control at 10 μg/ml for 2 h. Endogenous peroxidase was quenched using 0.3% H_2_O_2 _(Sigma-Aldrich Co. Ltd., St. Louis, MO) in 0,5% normal rabbit serum. After washing in PBS, sections were incubated with biotinylated mouse-adsorbed rabbit anti-rat IgG for 30 min. After washing, slides were treated with the avidin-biotin-complex (ABC)-horseradish peroxidase (HRP) reagent for 30 min. followed by HRP substrate NovaRed treatment. Slides were counterstained with haematoxylin (Vector Laboratories), dehydrated with alcohol, cleared with xylene, and mounted with microscopy Entellan (Merck).

### Fluorescent immunohistochemistry

Slides were acetone fixed and blocked in 10% normal rabbit serum for 15 min. Slides were drained and incubated with purified mAb 2A11 or the rat isotype control at 10 μg/ml for 1 hour. After washing in PBS, sections were incubated with Alexa 568 labelled goat anti rat IgG (Molecular probes) for 1 hour. After washing sections were incubated with FITC conjugated anti-mouse CD11b for 1 hour. Slides where washed and mounted in mounting medium containing DAPI (Vectashield).

### Measurements of colonic cytokines

Frozen colonic tissue was homogenized on ice in Greenberger Lysis Buffer (150 mM NaCl, 15 mM Tris, 1 mM MgCl·6H_2_O, 1 mM CaCl_2_, 1% Triton) with protease inhibitor cocktail from Roche (11697498001), pH 7.4, diluted 1:1 with PBS. Protein concentrations of IL-12, IFNγ, TNFα, IL-10, MCP-1 and IL-6 were measured in homogenates by cytometric bead array multiplex assay (BD Biosciences, San Jose, CA, USA) or using ELISA kits (R&D) according to manufacturer's protocol.

### Primary macrophage experiments

Thioglycollate-elicited peritoneal macrophages were isolated 4 days after intraperitoneal injection of 1 ml 4% (w/v) Brewer's thioglycollate medium (BD). 2.5 × 10^5 ^primary macrophages were plated in 24 well tissue culture plates with RPMI (Invitrogen) containing 50 IU/ml Penicillin, 50 μl/ml streptomycin and 2 mM L-glutamine at 37°C in 5% CO_2_. After 2 hours, non-adherent cells were removed by washing three times with medium. Diluted cecum content, LPS or zymosan (Molecular Probes) were added and incubated with the cells for 24 hours. Diluted cecum content was produced by suspending the contents of a mouse cecum in 50 ml PBS which was subsequently filtered over a 40 um filter and frozen at -20°C and used in a 1:1000 dilution. After 24 hours incubation no growth of micro-organisms was found in the wells and no cell death was observed by microscopic check for floating cells. Cytokine levels were determined using ELISA kits (R&D) according to manufacturer's protocol.

### Statistical analysis

All data are expressed as mean ± s.d. The statistical significance of the differences was evaluated using an unpaired *t*-test. Statistical significance was defined as *P *< 0.05.

## Results

### Dectin-1 localisation in mouse intestine

**Figure 1:**
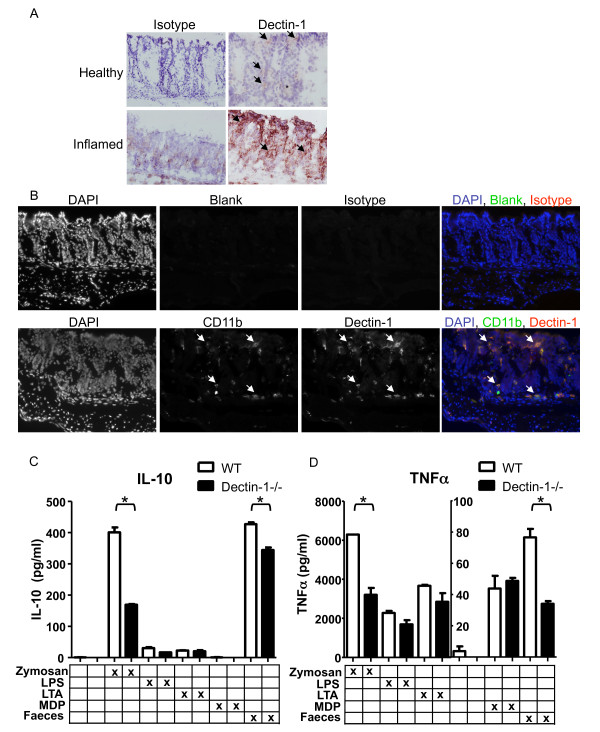
**Dectin-1 expression in colon and its involvement in macrophages activation after incubation with mouse faeces**. **A) **Dectin-1 staining (Nova Red) of WT colon using 2A11 on frozen sections. Sections were counterstained with haematoxylin. Representative image of un-inflamed tissue shows some dectin-1 expressing cells (arrows) in the mucosa. Representative image of inflamed colon after 7 days of DSS induce colitis showing an increase in dectin-1 expressing cells. The antibodies seemed to stick more to the inflamed tissue as is shown with the isotype control staining. **B) **Fluorescent immunohistochemistry showing DAPI, CD11b and dectin-1 staining of healthy intestine showing that dectin-1 expressing cells also express CD11b suggesting its macrophages, dendritic cells and neutrophils in the intestine that express dectin-1 (arrows). TNFα **(C) **and IL-10 **(D) **protein concentrations were measured in the supernatants of thioglycollate elicited macrophages isolated from WT C57BL/6 (white bars) or C57BL/6 dectin-1-/- (black bars) mice. Macrophages were incubated for 24 hours at 37°C with medium, zyomsan (0.5 particles/cell), LPS (100 ng/ml), LTA (5 ug/ml), MDP (10 ug/ml) or supernatants of mouse faeces. These data are representative of three independent experiments done in duplicate, error bars indicate the SD. A *t*-test was used for statistical analysis. *, p < 0.05.

We started our investigation by confirming that dectin-1 is located in the large intestine of mice. Immunohistochemical staining showed that dectin-1 is expressed in the lamina propria of the mouse colon (Figure 1A). As expected dectin-1 deficient mice did not show staining for dectin-1 in the intestine (data not shown). During DSS induced colitis the number of dectin-1 expressing cells in WT animals increased dramatically, suggesting an interaction with the intestinal microbiota under inflammatory conditions (Figure 1A). Fluorescent double staining showed that the main cells expressing dectin-1 in the intestine where also positive for CD11b i.e. macrophages, dendritic cells and neutrophils (Figure 1B).

### Dectin-1 mediated cytokine responses towards intestinal microbiota

To determine if dectin-1 is able to respond towards fungal or food components in mouse faeces we used WT thioglycollate-elicited peritoneal macrophages which are known to express dectin-1 [29] and compared their cytokine responses with those isolated from dectin-1 deficient mice (Figure 1C and 1D). As a positive control we incubated these cells with zymosan (a β-glucan containing particle and known ligand for dectin-1). Dectin-1 deficient macrophages showed a 50% reduction in both TNFα and IL-10 response after 24 hour incubation with zymosan. We also incubated macrophages with bacterial components like LPS, LTA and MDP which are not thought to interact with dectin-1 and indeed there were no differences in TNFα and IL-10 responses between WT and dectin-1 deficient macrophages (Figure 1C and 1D) showing that dectin-1 deficient macrophages were selectively deficient in dectin-1 specific responses and developed normally. Diluted mouse faeces induced IL-10 (Figure 1C) and TNFα (Figure 1D) production in WT macrophages. Faeces contains a whole array of various stimuli and our mouse faeces seems to contain components that can induce a high IL-10 response but does not stimulate TNFα production as much as zymosan or LPS do.

IL-10 and TNFα responses depend on the combination of pattern recognition receptors that are triggered. It has been shown that different bacteria are able to induce completely different cytokine profiles and can even work against each other [30,31]. The difference in magnitude of IL-10 and TNFα induced by faeces compared to zymosan is very likely due to different cell activation by the components, however a role for dectin-1 in the responses is clear in both stimuli since in dectin-1-/- macrophages these cytokine responses were significantly reduced, suggesting that dectin-1 deficient macrophages lack a response towards the fungal or food components found in the mouse intestinal microbiota (Figure 1C and 1D). The results shown are the response towards WT faeces, the same results were found when using dectin-1-/- faeces which suggests there are no differences in the intestinal microbiota between these mice.

A yeast classified as one of the *Rhodotorula *species was the only fungal component cultured consistently in faeces of both dectin-1-/- and WT mice (data not shown). *Rhodotorula sp*. can be found in faeces [32,33] and are considered non pathogenic [34]. *Rhodotorula sp*. are not considered to be medically important which made it impossible for us to further determine the exact species on our premises. Nonetheless these data show that dectin-1 could be involved in responses towards the fungal microbiota found in mouse intestine.

### DSS induced colitis in dectin-1 deficient mice

Next, we tested the effect of dectin-1 deficiency in an experimental model of DSS colitis. DSS is widely used as an inducer of inflammation in the intestine. It causes damage to the epithelial lining of the intestine which increases the interaction of the microbiota with the intestinal immune system, leading to an acute inflammation mainly involving innate immune cells [35]. Since dectin-1 is expressed in the myeloid compartment of the mouse intestine and is up-regulated during colitis (Figure 1A and 1B) and the lack of dectin-1 leads to reduced production of TNF-α and IL-10 production by macrophages we hypothesised that dectin-1 deficient mice would develop less inflammation after inducing DSS colitis.

**Figure 2:**
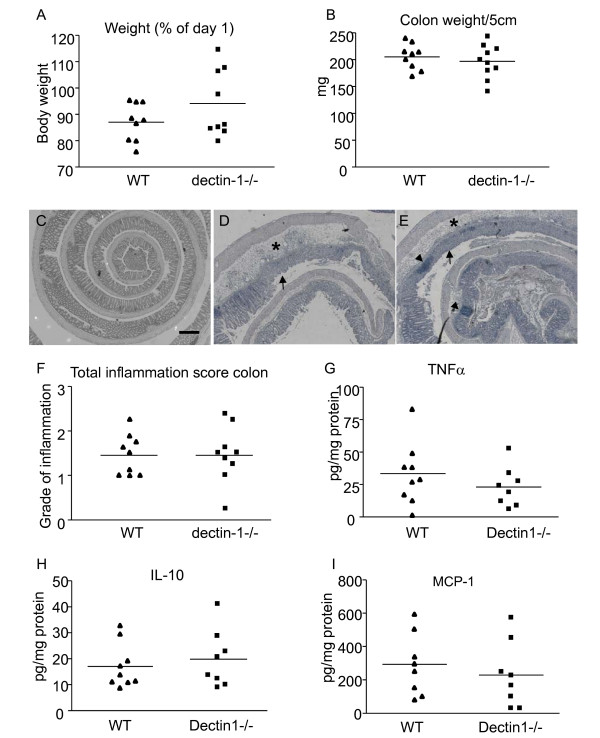
**Dectin-1 involvement in DSS induced colitis**. Mice were given 1.5% DSS in their drinking water for 7 days. **A) **Bodyweights were measured as indication of disease severity in this model, weights are shown as percentage of weight on day seven compared to day one. **B) **Colons were weighed as an indication of inflammation, shown as weight per 5 cm colon. F) Longitudinally divided colons were rolled, fixed and embedded in paraffin for routine histology. Colons were evaluated by an experienced pathologist and graded from 0 to 4 points as indicated in table 1. The total inflammation score was determined by the average score of the following criteria: area involved, the number of follicle aggregates, oedema, fibrosis, hyperplasia, erosion/ulceration, crypt loss and infiltration of granulocytes and mononuclear cells. **C) **Representative picture of healthy colon. **D) **Representative picture of WT colon after 7 days of DSS induced inflammation. **E) **Representative picture of dectin-1-/- colon after 7 days of DSS induced inflammation. In inflamed colon a large amount of cell infiltration was found in both the mucosa and submucosa (asterisk), areas of cryptloss were clear (arrow) and we also found gut associated lymphoid tissue (arrowheads). Bar, 200 um. **G) **TNFα protein concentrations in colon lysates **H) **IL-10 protein concentrations in colon lysates **I) **MCP-1 protein concentrations in colon lysates. The sample means are indicated with a line. A *t*-test was used for statistical analysis. *, p < 0.05, n = 10.

To test this we induced DSS colitis and after 7 days mice lost 5-20% weight due to disease but no significant differences were found in weight loss between dectin-1 deficient and WT mice (Figure 2A). No differences in spleen weight were found (data not shown). Colon weight, which is a measure of colon inflammation and increases due to cell infiltration and oedema, did not show significant differences between the two groups either (Figure 2B). Histological scoring showed that both WT and dectin-1 deficient mice had equal severe inflammation in the intestine with crypt loss, crypt erosion, ulceration, oedema and infiltration of both monocytes and granulocytes. No significant differences were found in these parameters in intestinal inflammation (Figure 2F). Representative pictures of healthy colon, WT inflamed colon and dectin-1-/- inflamed colon are shown in Figure 2C-D. We also analysed cytokine levels in mouse colons and serum and were able to measure TNF-α, MCP-1 and IL-10 in the colon lysates. Colons of mice without induced inflammation did not contain measurable cytokine levels (data not shown) and no significant differences were found between the two groups in inflamed colons (Figure 2G-I). Unexpectedly, these data show that DSS colitis in dectin-1 deficient mice develops the same as in WT mice.

### *Helicobacter hepaticus *induced colitis in dectin-1 deficient mice

**Figure 3:**
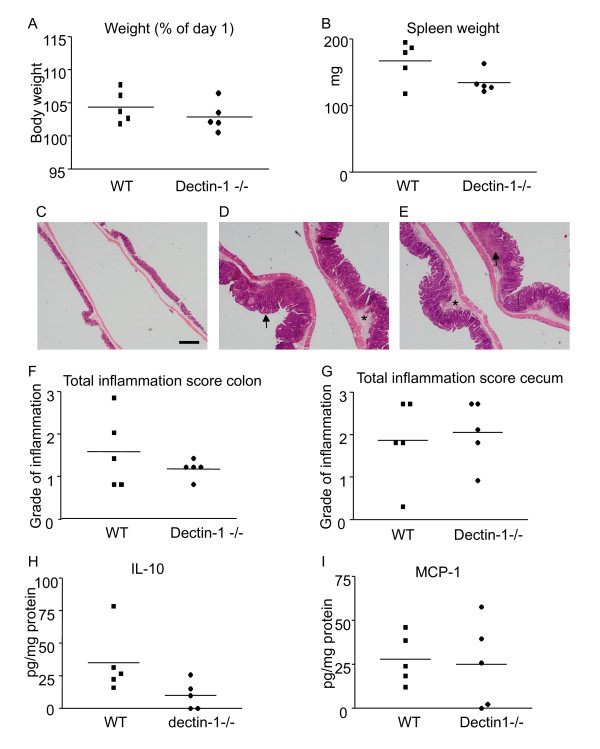
**Dectin-1 involvement in *H. hepaticus *induced colitis**. Mice were inoculated intragastrically with *H. hepaticus *and received weekly i.p. injections of 1 mg anti-IL-10R for the duration of four weeks. **A) **Bodyweights were measured, weights are shown as percentage of weight on day 28 compared to day one. Disease severity was measured using **B) **spleen weight F) colon total inflammation score and G) cecum total inflammation score. **C) **Representative picture of healthy cecum. **D) **Representative picture of WT cecum after *H. hepaticus *induced colitis. **E) **Representative picture of dectin-1-/- cecum after *H. hepaticus *induced colitis. In inflamed cecum a large amount of cell infiltration was found in both the mucosa (arrow) and submucosa (asterisk). Bar, 500 um. H) IL-10 protein concentrations in colon lysates I) MCP-1 protein concentrations in colon lysates. The sample means are indicated with a line. A *t*-test was used for statistical analysis. *, p < 0.05, n = 5.

Since DSS induced colitis did not show a role for dectin-1 in intestinal inflammation we tested another colitis model that is microbiota driven. *H. hepaticus *infected C56BL6 mice that received I.P injections with anti-IL-10 receptor antibodies develop a chronic typhlocolitis over the course of four weeks which is T cell dependent with a mixed Th1/Th17 response [27]. The mechanisms through which *H. hepaticus *is able to induce chronic typhlocolitis are still unclear [36]. Results were variable among different animals and no significant differences were found in weight, spleen weight, colon and cecum pathology (Figure 3A, B, F and 3G). Representative pictures of healthy cecum, WT inflamed cecum and dectin-1-/- inflamed cecum are shown in Figure 3C-E. Levels of inflammatory cytokines were measured in lysed colon and serum samples, only IL-10 and MCP-1 were above the detection limit in the lysed colon, but no consistent or significant differences were found between WT and dectin-1 deficient animals (Figure 3H and 3I).

## Discussion

The intestinal immune system is shaped by its interaction with the microbiome and vice versa [1,37-39]. Dectin-1 is a PRR able to influence innate and adaptive immune responses upon recognition of fungi [40]. Indeed our *in-vitro *data show that faeces from our mice are able to induce dectin-1 dependent cytokine responses. Our data suggest that the luminal flora or food components from our mice are able to interact with dectin-1 and stimulate IL-10 and TNF-α production by macrophages. Importantly however, our data indicate that dectin-1, does not play a vital role in experimental colitis in mice.

Crohn's disease patients have been found to produce antibodies against fungal glycocarbohydrates including β-glucans and mannans [16,17]. *C. albicans *is a suspected immunogen for these antibodies [41,42] and as a major receptor for *C. albicans *[29,43], dectin-1 is likely to be important in immune responses involving patients with an intestinal *C. albicans *infection. Indeed, *C. albicans *has been described to aggravate inflammation in DSS induced colitis [44]. However, mice are not naturally infected with *C. albicans *and we did not find Candida species in our mice. We found a fungus of the Rhodutorula spp in the stools of our mice. Besides their possible presence in faeces, these fungi are often found in humid environments like bathrooms and soil and are not considered to be pathogenic or play a role in colitis [33].

We used two different colitis models to determine if dectin-1 plays a role in the progression of intestinal inflammation. When adding DSS to the drinking water for 7 days, mice develop an acute inflammation which is mainly driven by the innate immune system as T and B cell deficient mice like RAG-/- and SCID animals also develop colitis after feeding DSS [35,45]. The second model we used was based on infection with *H. hepaticus *in mice that received antibodies directed at the IL-10 receptor. In these mice inflammation develops over a course of four weeks and both innate and adaptive immune responses are involved in this chronic model of colitis [28]. In both models, dectin-1 deficient mice did not show any substantial or consistent differences in weight, colon inflammation, systemic inflammation and cytokine responses suggesting that the course of inflammation is the same for WT and dectin-1-/- mice in these models. This was surprising since in-vitro experiments showed that dectin-1 deficiency had significant effects on cytokine responses towards the mouse faeces (Figure 1). It may be that during intestinal inflammation other PRRs compensate for the lack of dectin-1; several other receptors are known to recognise fungi including TLRs [46,47], mannose receptor [29,48], dectin-2 [49] and DC-SIGN [50] and its mouse homologue SIGN-R1 [51]. Since bacteria are the dominant bowel inhabitants and only about 1% of the intestinal microbiome consists of fungi [14], it may also be possible that dectin-1 involvement in intestinal inflammation is overwhelmed by responses towards the bacterial component.

Various PRR have been shown to play an important role in human IBD and dectin-1 has been shown to co-signal with TLR2 and TLR6 for the production of various pro-inflammatory cytokines [19,20,22]. Clearly, although our data suggest dectin-1 signalling is redundant in intestinal inflammation TLR2 and/or TLR6 deficiency does affect experimental colitis via separate mechanisms indicating that dectin-1 deficiency does not seem to affect TLR signalling. This was also indicated by our observation of normal responses to TLR ligands other that dectin-1 in deficient cells. Indeed, a mutation found in human dectin-1 which leads to partial dectin-1 deletion has been shown not to be involved in IBD [26]. It is likely that dectin-1 becomes more relevant when the fungal burden in the intestine increases, for instance due to antibiotic treatment or infection.

## Conclusions

Our *in-vitro *data suggest that dectin-1 is able to induce a cytokine response towards mouse faeces, however dectin-1 deficiency in mice does not affect the course of inflammation in two models of experimentally induced colitis suggesting that dectin-1 signalling is redundant in experimental colonic inflammation induced by either DSS or *H. hepaticus *in mice.

## Abbreviations

CLR: C-type lectin-like receptors; DSS: Dextran Sulphate Sodium; IBD: Inflammatory bowel diseases; LPS: Lipopolysaccharide; LTA: Lipoteichoic acid; PRR: Pattern recognition receptor; TLRs: Toll-like receptors; WT: Wild type.

## Competing interests

The authors declare that they have no competing interests.

## Authors' contributions

SH participated in the design of the study, experimental work and drafting the manuscript. AO participated in the helicobacter experiments. JR participated in the immunohistochemistry analysis and did the pathology scoring. SD and AV participated in DSS colitis experiments. SG and WJ participated in the design of the study and drafting the manuscript. All authors read and approved the final manuscript.

## Pre-publication history

The pre-publication history for this paper can be accessed here:

http://www.biomedcentral.com/1471-230X/12/33/prepub
